# Lubricin protects against cartilage degeneration following anterior cruciate ligament transection in rats

**DOI:** 10.1038/s41598-026-45349-9

**Published:** 2026-04-20

**Authors:** Sydney J. Womack, Erica J. Secor, Sophie R. Nelissen, Aiyana G. Fortin, Ashley M. Schiller, Marshall J. Colville, Lawrence J. Bonassar, Matthew J. Paszek, Heidi L. Reesink

**Affiliations:** 1 Department of Clinical Sciences, Cornell University College of Veterinary Medicine, Ithaca, NY USA; 2 Department of Biomedical Sciences, Cornell University College of Veterinary Medicine, Ithaca, NY USA; 3 Meinig School of Biomedical Engineering, Cornell University, Ithaca, NY USA; 4 Smith School of Chemical and Biomolecular Engineering, Cornell University, Ithaca, NY USA; 5 Sibley School of Mechanical and Aerospace Engineering, Cornell University, Ithaca, NY USA; 6 Department of Surgical and Radiological Sciences, School of Veterinary Medicine, University of California, Davis, Davis, CA USA; 7 Department of Biomedical Engineering, University of California, Davis, Davis, USA

**Keywords:** ACLT, Sex differences, Lubricin, Histology, Hyperalgesia, OA therapeutic, Diseases, Medical research, Rheumatology

## Abstract

Despite the growing prevalence of post-traumatic osteoarthritis (OA), early and effective treatment options to delay surgical intervention remain limited. Lubricin is a naturally occurring glycoprotein produced by articular chondrocytes and synovial fibroblasts that functions as the principal boundary lubricant in joints. Recently, a recombinant lubricin-like glycoprotein (rhLub) with codon optimized mucin domain was reported to have a sustained residence time (> 6 weeks) following injection into the knee joint of healthy rats. To evaluate outcomes of rhLub injection, skeletally mature Sprague-Dawley rats of both sexes (*n* = 18) underwent bilateral anterior cruciate ligament transection (ACLT) to initiate post-traumatic OA, followed by three intra-articular injections of 25 µL of rhLub (1.3 mg/mL) or phosphate-buffered saline (PBS) at weekly intervals beginning 1 week post-operatively. Pain sensitization was evaluated weekly throughout the 12-week study with weightbearing and pressure application measurement (PAM). Frontal plane histologic sections of the medial femorotibial joint compartment were scored using Osteoarthritis Research Society International criteria. Injections of rhLub were well-tolerated. rhLub injections protected against cartilage degeneration and improved histologic scores in male rats. Mechanical hyperalgesia and cartilage degeneration scores were greater in female rats compared to males, irrespective of treatment. This study suggests that intra-articular rhLub therapy may be most efficacious in mild-to-moderate disease.

**Supplementary Information:**

The online version contains supplementary material available at 10.1038/s41598-026-45349-9.

## Introduction

Knee osteoarthritis (OA) affects millions of people worldwide, is characterized by pain and stiffness, and results in a decreased quality of life^1^. Current therapeutic options are limited to lifestyle changes such as weight loss and exercise, opioids or non-steroidal anti-inflammatory drugs (NSAIDs) for pain management, and injectables such as hyaluronic acid or corticosteroids, with total knee replacement as a last-resort option^2^. Systemic oral drugs like NSAIDs and opioids are not recommended for chronic use due to adverse side effects, with NSAID use in older patients associated with increased cartilage lesions compared to non-users^3^. Intra-articular corticosteroids are a disease-modifying therapeutic option that can provide temporary relief^4^, but long-term administration is advised against due to the potential for increased pain, stiffness, and decreased functional ability with repeated injections over 6 months^5,6^. Therapies for mild-to-moderate OA that can reliably prevent joint degeneration and improve pain with few adverse side effects are lacking.

Viscosupplements and tribosupplements, inspired by molecules naturally occurring in joint fluid, hold promise as pain-reducing and disease-modifying OA therapeutics. Synovial fluid is a highly viscous and lubricating fluid formed as an ultrafiltrate of plasma enriched with hyaluronic acid (HA), lubricin, and other proteins, including albumin and immunoglobulins^7^. In addition to its mechanical role, synovial fluid transports nutrients to joint tissues, including articular cartilage. Synovial fluid viscosity, provided predominantly by high molecular-weight HA^8^, also contributes to its chondroprotective properties. Lubricin is a high molecular-weight glycoprotein with anti-adhesive^9^ and anti-inflammatory^10^ properties that, through synergistic activities with HA and other molecular components^11^, enables low friction synovial joint lubrication.

With the protective and lubricating effects of native synovial fluid, HA and lubricin have both been proposed as potential intra-articular therapies. Despite the existence of multiple injectable HA formulations currently approved as treatments for OA, reports on their efficacy are conflicting. However, HA injections continue to be widely used clinically with moderate pain-relieving effects reported^12^. HA and lubricin both contribute to synovial fluid’s lubricating characteristics through different, but synergistic mechanisms^13,14^. Early iterations of injectable recombinant and purified human lubricin were shown to be effective in reducing cartilage degeneration in rat injury models, including following anterior cruciate ligament transection (ACLT)^15,16^. The rhLub reported here is an engineered biolubricant distinct from native lubricin that closely mimics lubricin’s structure and function. rhLub retains the lubricating properties of native lubricin through its shared mucin domain^17^. However, where lubricin’s mucin domain is composed of a variable number of imperfect tandem repeats, rhLub was engineered to have exactly 59 perfect KEPAPTTP repeats using a combination of codon scrambling and optimization to achieve stability and high titers in mammalian expression systems. rhLub shares N- and C-termini with native lubricin, recapitulating lubricin’s ability to assemble into a polymer brush on the surface of articular cartilage, and is manufactured using a novel production strategy in a human cell line. This production process more closely recapitulates the native glycosylation of human lubricin^18–20^. Recombinant lubricin’s functionality in vivo is dependent on its manufacturing process^21^, and with the unique strategies used to develop rhLub, this study is the second to investigate its potential therapeutic impact in an OA animal model^22^. The objective of this study was to evaluate the safety and efficacy of three repeated rhLub injections in rat knee joints of both sexes post-ACLT with histologic structural outcomes as the primary measure and assessment of pain sensitization as a secondary measure.

## Results

### There were no functional deficits observed in the rhLub-injected limb in rats of either sex

An overview of the study design can be found in Supplementary Fig. S1. Weightbearing results did not vary as a function of sex or time post-surgery, which was not unexpected given the bilateral nature of the model. Weightbearing asymmetry was not present between treatment legs, with animals distributing equal weight on rhLub-injected limbs relative to total weightbearing (50.4%, *n* = 18) (Supplementary Fig. S2). Imbalance was present as a function of laterality with rats bearing more weight on left (51.6%) compared to right (48.4%) limbs (*p* < 0.0001, *n* = 18). Variance in the immediate post-operative period and in the last three weeks of the study was greater than in the middle portion of the experiment (weeks 4–8) for both male and female rats. Male rats (*n* = 9) gained an average of 113 ± 4.5 g (36% increase from week 0) and female rats (*n* = 9) gained an average of 44 ± 1.9 g (23% increase from week 0) over the 12-week period (Supplementary Fig. S3, Table S1). One female rat was euthanized 6 days post-operatively, prior to intra-articular injection, due to repeated suture removal and wound trauma in one limb. One male rat was excluded from analysis due to a subclinical suture-site abscess detected in the PBS-injected limb at necropsy.

### Female but not male animals developed progressive pain sensitization bilaterally over time compared to baseline

**Fig. 1:**
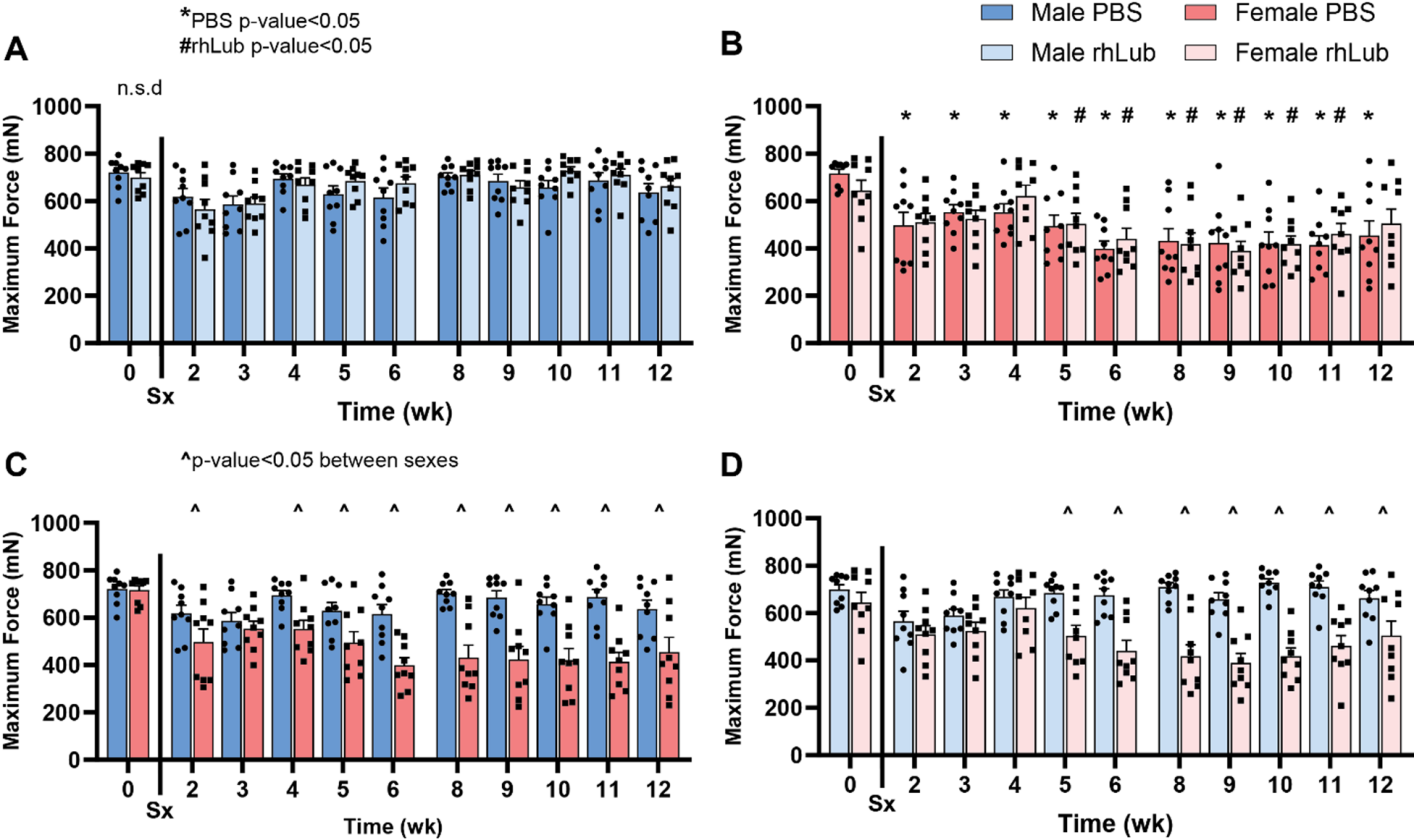
Mechanical hyperalgesia in rhLub- and PBS-injected limbs from male (*n* = 9) and female (*n* = 9) rats following anterior cruciate ligament transection (ACLT). Pressure application measurement (PAM) did not provide evidence for mechanical hyperalgesia as a result of rhLub treatment in either sex (**A**,**B**). Where male animals did not have sensitization at any point in the study (**A**), female animals had increased pain sensitization from baseline starting at week 2 in PBS-injected limbs and at week 5 in rhLub-injected limbs (**B**). Female rats had increased pain sensitization relative to male rats at weeks 2 and 4–12 in PBS-injected limbs (**C**) and weeks 5–12 in rhLub-injected limbs (**D**). Sx = surgery. *PBS *p* < 0.05, #rhLub *p* < 0.05, ^*p* < 0.05 between sexes.

Males did not show sensitization as measured via mechanical hyperalgesia in either limb compared to baseline for the duration of the study (Fig. 1A). Female animals showed increased pain sensitization in the PBS-injected limb in the immediate post-operative period at week 2 (497 ± 55 mN, *p* = 0.0002) and at weeks 3 (553 ± 30 mN, *p* = 0.02), 4 (553 ± 36 mN, *p* = 0.02), and 5 (494 ± 47 mN, *p* = 0.0002) compared to baseline (716 ± 18 mN) (Fig. 1B). Females showed persistent sensitization at weeks 6 (399 ± 28 mN, *p* < 0.0001) through 12 (454 ± 33 mN, *p* = 0.0001) in PBS-injected limbs and weeks 6 (440 ± 44 mN, *p* = 0.0009) through 11 (462 ± 43 mN, *p* = 0.005) in rhLub-injected limbs (Fig. 1B). Females had increased pain sensitization compared to males at week 2 (-121 mN, *p* = 0.02) and weeks 4 (-141 mN, *p* = 0.008) through 12 (-181 mN, *p* = 0.0007) in PBS-injected limbs and at weeks 5 (-181 mN, *p* = 0.0008) through 12 (-157 mN, *p* = 0.003) in rhLub-injected limbs (Fig. 1C and D). Baseline maximum force was not different between males (709 ± 14.1 mN) and females (680 ± 24.3 mN) (*p* = 0.5, *n* = 18) (Supplementary Tables S2, S3). For nearly every Osteoarthritis Research Society International (OARSI) subcomponent, female scores were either no different or greater than male scores (Table S1 Females tended to have increased significant cartilage damage and overall OARSI score compared to males, irrespective of treatment (*p* = 0.05 and 0.07, respectively) (Supplementary Fig. S4). Cartilage matrix loss at the surface was similar between males and females, but female animals had more severe cartilage degeneration that extended further into the cartilage than males independent of rhLub or PBS injection (*p* = 0.03) (Supplementary Fig. S4).

### rhLub-treated joints in male animals had improved histologic scores relative to contralateral PBS-treated joints

**Fig. 2:**
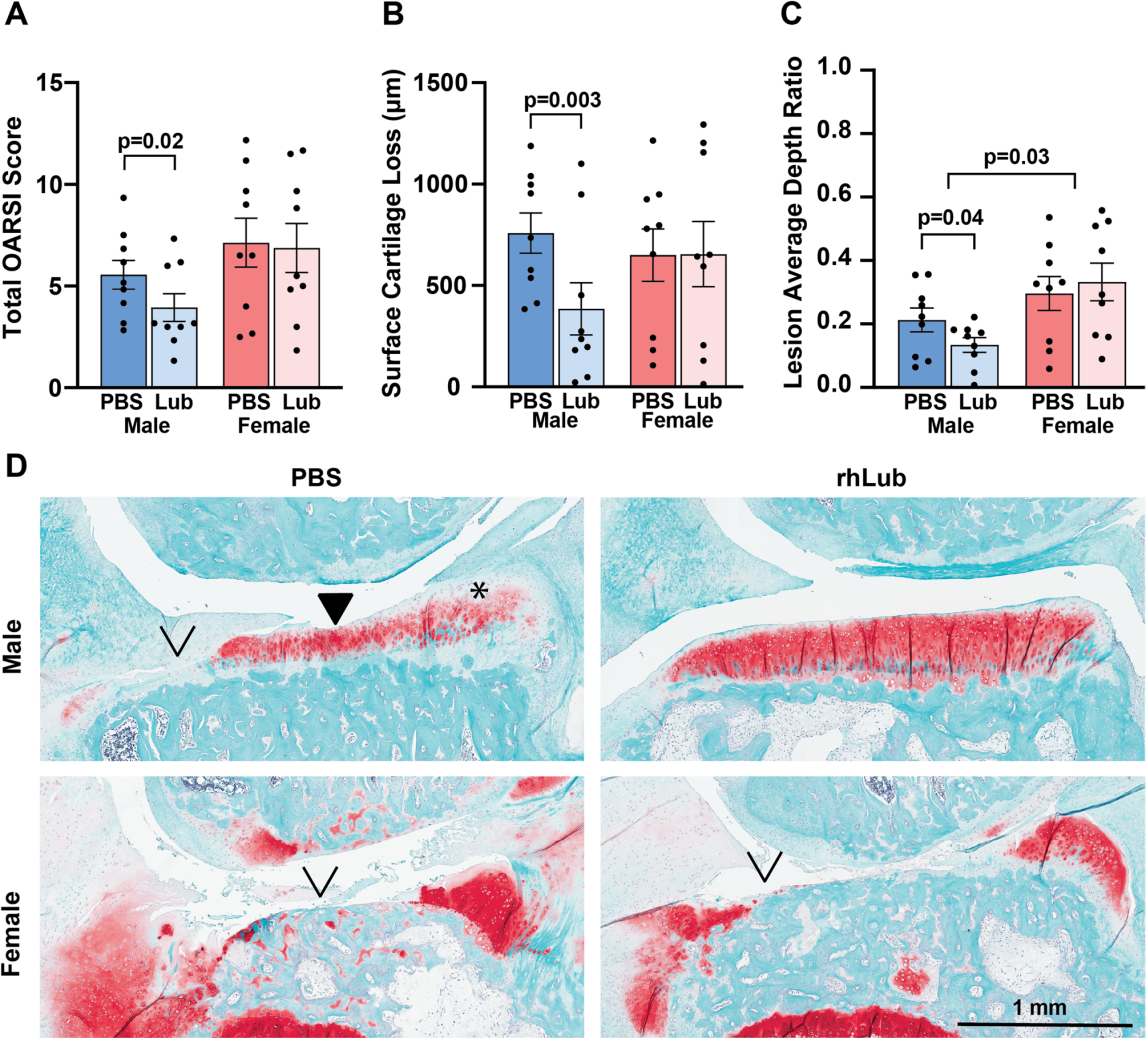
rhLub injection protected against cartilage degeneration in male but not female rats, and female rats had more severe cartilage loss compared to males. Total OARSI scores were lower in rhLub-injected joints from male rats (**A**). Male joints that received rhLub treatment had decreased cartilage matrix loss at the surface (**B**) with shallower lesions compared to PBS-treated joints (**C**). Lesion depth was greater in female compared to male animals independent of treatment (**C**). PBS-treated joints exhibited greater cartilage matrix loss at the surface (closed arrowhead ▲) and middle (open arrowheads ^) of the medial tibial plateau compared to rhLub-treated joints within-animal (**D**). The length of cartilage with degenerative changes, including loss of proteoglycan staining (asterisk *), fibrillation, and chondrocyte death was greater in PBS-treated compared to rhLub-treated joints (**D**). Overall cartilage degeneration (*p* = 0.03) and depth of lesions (*p* = 0.03) (SupplementaryFig. S4), characterized by matrix loss particularly at the middle and tidemark of the cartilage, was significantly greater in female compared to male joints regardless of treatment (**D**). Representative sections are taken from the mid-caudal frontal plane section.

In males, rhLub treatment resulted in improved histologic scores in 67% (6/9) of OARSI subcomponents, while females showed no differences in histologic scores as a function of treatment (Table 1). Overall OARSI score was lower in rhLub-injected knees in male rats (3.9 ± 0.81) compared to PBS-injected knees (5.6 ± 1.1, *p* = 0.02) (Fig. 2A). Joints that received rhLub intra-articular injection had decreased cartilage matrix loss at the surface (376 ± 165 μm, *p* = 0.003) and through the midzone of the medial tibial cartilage (49 ± 58 μm, *p* = 0.03) compared to PBS treatment at the surface (750 ± 206 μm) and midzone (161 ± 90 μm) in males (Fig. 2B, Supplementary Fig. S4). In male rhLub-treated joints, 41% of the medial tibial cartilage was affected by degenerative changes (914 ± 249 μm, *p* < 0.0001) compared to 57% in PBS-treated joints (1516 ± 1005 μm) (Supplementary Fig. S4). Subchondral bone in the medial tibia was protected from degenerative changes in rhLub-treated joints (0.44 ± 0.16, *p* = 0.01) relative to PBS-treated joints (0.98 ± 0.98) in males (Supplementary Fig. S4). Lesion depth ratios were lower in rhLub-treated joints (0.13 ± 0.05, *p* = 0.04) compared to PBS treatment (0.21 ± 0.18) in males (Fig. 2C). Attenuation of degenerative histologic changes, including reduced fibrillation, decreased cartilage matrix loss, and increased proteoglycan staining, was noted in rhLub-treated limbs (Fig. 2D). Upon application of a Bonferroni correction for the post-hoc between-treatment comparisons within males, all values remained significant at the α < 0.05 level except the cartilage matrix loss at midzone (*p* = 0.07) and the lesion depth ratio (*p* = 0.09).

**Table 1:** OARSI subcomponents.

	Male			Female			Sex
	PBS	rhLub	*p*-value	PBS	rhLub	*p*-value	*p*-value
#1a. Cartilage matrix loss at surface (0% depth) (µm) (†)	750 ± 206	376 ± 165	0.003*	650 ± 177	654 ± 184	1.0	0.6
#1b. Cartilage matrix loss at midzone (50% depth) (µm) (†)	161 ± 90	49 ± 58	0.03*	241 ± 104	214 ± 93	0.6	0.2
#1c. Cartilage matrix loss at tidemark (100% depth) (µm) (†)	36 ± 29	21 ± 22	0.4	64 ± 45	56 ± 40	0.7	0.4
#2. Cartilage degeneration score	4.1 ± 0.87	3.1 ± 0.79	0.07	5.7 ± 1.1	5.7 ± 0.96	0.9	0.03*
#3. Total cartilage degeneration width (µm) (†)	1129 ± 79	817 ± 74	0.004*	1197 ± 63	1225 ± 60	0.7	0.13
#4. Significant cartilage degeneration width (µm) (†)	260 ± 43	121 ± 23	0.07	437 ± 52	405 ± 44	0.6	0.05
#5. Zonal depth ratio of lesions	0.21 ± 0.06	0.13 ± 0.05	0.04*	0.30 ± 0.08	0.33 ± 0.09	0.3	0.03*
#6. Osteophytes	0.46 ± 0.28	0.39 ± 0.25	0.5	0.5 ± 0.26	0.30 ± 0.17	0.2	0.9
#7. Calcified cartilage and subchondral bone damage score	0.98 ± 0.33	0.44 ± 0.16	0.01*	0.96 ± 0.38	0.91 ± 0.30	0.8	0.4
#8. Total combined OARSI score	5.6 ± 1.1	3.9 ± 0.81	0.02*	7.1 ± 1.5	6.9 ± 1.3	0.6	0.07

Data represented as mean ± standard error of the mean (SEM), with an α < 0.05 significance between treatments. (†) represents score that is normalized to total medial tibial cartilage length.

### rhLub treatment was chondroprotective at the individual level in males

**Fig. 3:**
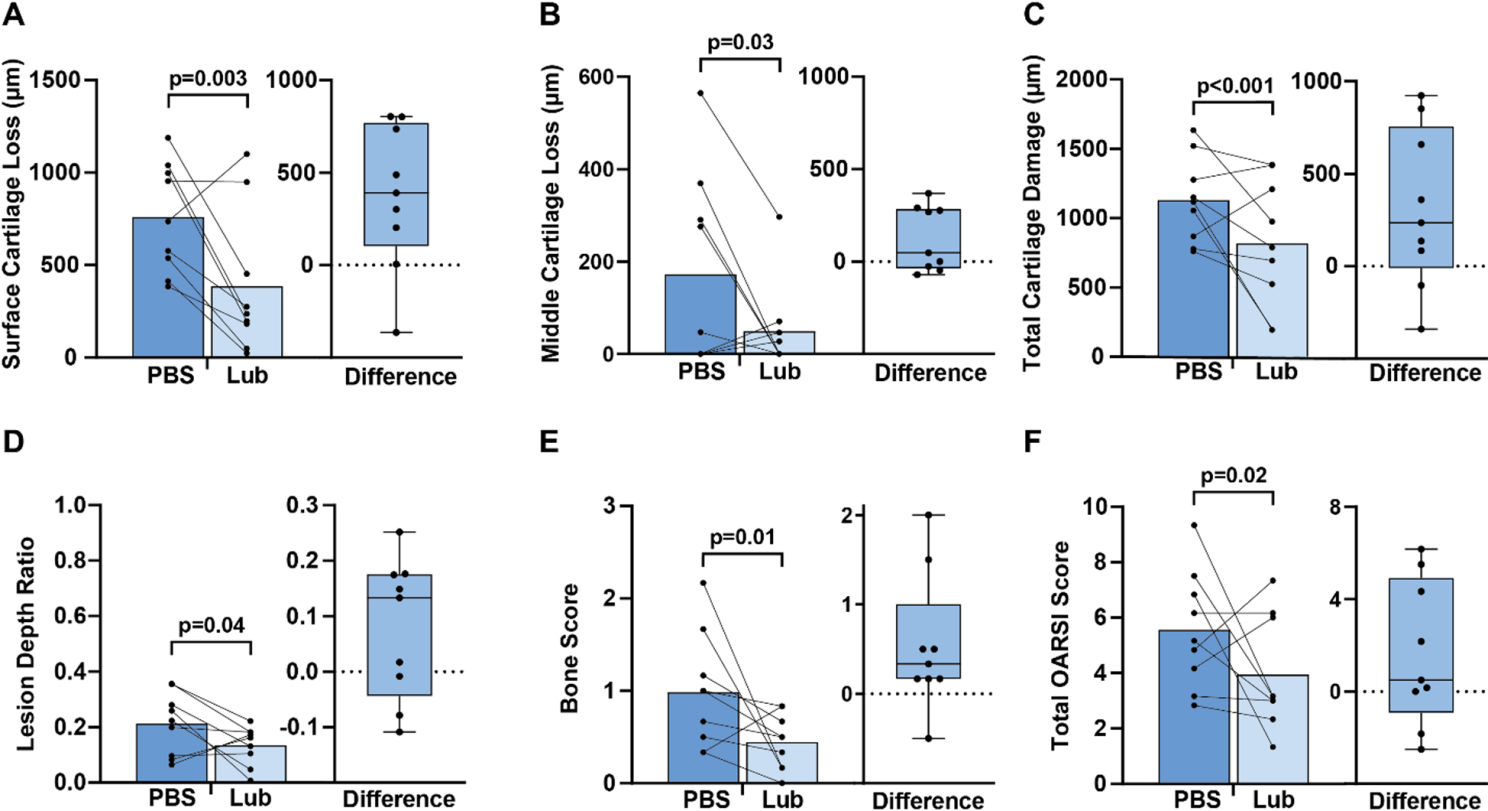
In the majority of male rats, rhLub intra-articular injection protected against degenerative joint changes compared to PBS injection within different knees in the same rat. rhLub treatment resulted in uniform improvement in male joints, with no differences in female rats. Medial tibial cartilage matrix loss at the surface (**A**) and middle (**B**) of the plateau, as well as total cartilage damage including subtle and overt degenerative changes (**C**), was reduced following rhLub treatment compared to contralateral, PBS-injected knees in most animals. Lesions present in rhLub-injected knees were shallower than lesions in PBS-injected knees in the majority of rats (**D**). Excepting one score, bone remodeling and degenerative changes (**E**) were reduced following rhLub treatment compared to contralateral, PBS-injected knees. Total OARSI score was lower in rhLub-injected knees in most rats (**F**). Lines connect points representing scores from each limb within the same animal. Difference plots represent rhLub scores subtracted from PBS scores within the same animal.

Histologic scores indicated reduced joint damage in rhLub-treated compared to PBS-treated joints in at least 56% (5/9) of individual animals for 8 out of 9 OARSI subcomponents. For four of the subcomponents, histologic damage scores were reduced in the rhLub-treated joint in at least 78% (7/9) of individuals. There was an average 374 ± 131 μm (51%) less medial tibial cartilage loss at the surface in rhLub- compared to PBS-injected joints within the same individual (*p* = 0.003) (Fig. 3A). At the middle cartilage zone, there was 123 ± 58 μm (89%) less medial tibial cartilage loss in rhLub-injected joints within the same individual (*p* = 0.03) (Fig. 3B). The length of total degenerative cartilage changes in the medial tibia was 312 ± 143 μm (54%) less in rhLub- as opposed to PBS-treated joints within the same individual (*p* < 0.001) (Fig. 3C). Medial tibial cartilage lesions were shallower in rhLub-injected limbs, with the depth ratios an average of 0.08 ± 0.04 (39%) less than in PBS-treated joints within the same animal (*p* = 0.04) (Fig. 3D). Medial tibia bone scores were on average 0.074 ± 0.24 points (58%) lower in joints treated with rhLub as compared to PBS within the same individual (*p* = 0.01) (Fig. 3E). Overall OARSI scores were 1.6 ± 1 (46%) points lower in rhLub-treated joints than in PBS-treated joints within the same individual (*p* = 0.02) (Fig. 3F). Subcomponent scores at the individual level in females were unchanged.

## Discussion

Here, we report the first investigation into the efficacy of a novel intra-articular recombinant human lubricin-like glycoprotein (rhLub) in a rodent post-traumatic osteoarthritis (OA) model. We demonstrate that intra-articular injection of rhLub is protective against cartilage degeneration in male rats after anterior cruciate ligament transection (ACLT). Specifically, intra-articular injection of rhLub protected against medial tibial cartilage loss, cartilage matrix degeneration (e.g., proteoglycan loss, matrix loss, chondrocyte death, and lesion depth), and bone degeneration. rhLub treatment did not cause any adverse functional or pain outcomes in males or females. Interestingly, females displayed more severe functional and structural outcomes than males, irrespective of treatment. Ultimately, this study shows attenuation of mild-to-moderate cartilage degeneration post-intra-articular injection with a bioengineered recombinant lubricin-like glycoprotein in rats after ACLT^19^.

Importantly, recent work has shown that function of recombinant lubricin varies based on its production technique, even when made in the same cell line^21^. This is the second study to investigate the in vivo functionality of a recombinant lubricin-like therapeutic produced in human cells using a mucin codon optimization strategy combined with a novel manufacturing process and cation exchange purification strategy^22^. Like lubricin purified from synovial fluid^23^ and recombinant lubricin produced in synoviocytes^9^ or Chinese Hamster Ovary (CHO) cells^24^, rhLub retains a lubricating ability comparable to native synovial fluid^19^. Prior studies support the chondroprotective effects of purified and recombinant lubricin in animal models^16^. Both genetic restoration of proteoglycan 4 (*Prg4*)/lubricin and intra-articular injection of lubricin protect against cartilage fibrillation and glycosaminoglycan loss in rodent models^25,26^. rhLub shares the N- and C-terminal domains, as well as the mucin-like domain, responsible for its cartilage-binding and lubricating abilities, respectively, with native lubricin^27^. Indeed, rhLub molecules self-assemble on cartilage, and retain the lubricating and tissue binding properties of native lubricin^19,28^. rhLub is manufactured in Human Embryonic Kidney (HEK-293 F) suspension cells. Compared to CHO cells, recombinant proteins produced from HEK-293 cells have glycosylation profiles similar to native human cells^29^. rhLub has a prolonged half-life of 46 days post-injection in rat knee joints^19^.

Because lubricin is the primary boundary lubricant in synovial fluid, loss of lubricin is correlated with an increased coefficient of friction in synovial fluid^30^. Similarly, lubricin administration decreases the friction coefficient of joint fluid both by aggregating on the cartilage surface and interacting with articular cartilage in its soluble form^24^. With low blood supply to the articular cartilage, hypoxic conditions are common^31^. In healthy joints, hypoxia inducible factors 1 and 2 α (Hif1α, Hif2α) can protect against hypoxia-induced chondrocyte apoptosis and degradation of the extracellular matrix (ECM) in the homeostatic pathway and also contribute to chondrocyte hypertrophy and differentiation in the degradation pathway^31^. In addition to the anti-adhesive effects afforded by its physical structure^9^, *Prg4* indirectly downregulates Hif1α and Hif2α, preventing cartilage turnover and ECM breakdown^31^. In this study, rhLub was protective of post-traumatic OA-associated medial tibial subchondral bone remodeling in male rats. While the surface chondroprotective effects of lubricin are well-characterized, the improved bone scores with rhLub treatment may indicate that articular cartilage integrity contributes to protection of the underlying subchondral bone. Notably, while some previous work^15,32^ has not found an effect of intra-articular lubricin treatment on bone, lubricin but not HA or steroid treatment attenuated subchondral bone density loss in *Prg4*-deficient mice, suggesting that lubricin could have a direct bone-protective effect in post-traumatic OA^33^. Lubricin is normally produced by superficial zone chondrocytes and synoviocytes^34^ but not osteocytes^35^.

Prior assessment of a synthetic polymer boundary lubricant resulted in more pronounced reductions in static weightbearing post-ACLT compared to phosphate-buffered saline (PBS) treatment, demonstrating the potential to detect pain sensitization in a unilateral injury model^36^. In the current study, no pain sensitization was noted in rhLub-treated limbs as compared to PBS-treated limbs measured either via static weightbearing or pressure application measurement (PAM). As Sprague-Dawley rats are an outbred rat strain, a bilateral injury model was chosen to investigate the effects of rhLub treatment while controlling for the effect of the individual rat. This experimental design allowed for individual-level analysis of treatment effects. rhLub treatment improved 8 out of 9 histologic subcomponent scores in most male rats. Bilateral, paired injury models were implemented under guidance of the “reduction” component of the 3R’s for animal use in biomedical research. To isolate any potentially mild effect of rhLub on pain sensitization and static weightbearing post-injury, future work with this recombinant lubricin could utilize unilateral injury and treatment models. rhLub is hypothesized to be a disease-modifying drug, rather than a primary analgesic. As such, while possible pain-reducing effects could be observed long-term, acute pain relief was not hypothesized to be an outcome of rhLub treatment. Notably, pain relief in preclinical OA models is most reliably observed after administration of primary analgesics such as NSAIDs^37^ and opioids^38^, which are known to have adverse long-term side effects^39–41^.

In early investigations of novel therapeutics in preclinical rodent animal models, intra-articular injections are often repeated to ensure sustained treatment residence times^42,43^. Several prior studies have utilized a similar dosing interval of once weekly injections for 3 weeks for evaluation of intra-articular lubricants^36,42^. In human medical practice, intra-articular injections are likely to be distributed at less frequent intervals due to slower metabolism and altered pharmacokinetics/pharmacodynamics^44,45^. Given rhLub’s therapeutic potential and prolonged residence time in the rat joint^19^, future studies could investigate a variety of intra-articular injection frequencies and dosages in preclinical models to optimize translation into the clinic.

To date, this is one of few studies that have investigated sex as a variable in rats with post-traumatic OA. One study investigated sex differences after medial meniscal transection in rats and noted increased osteophyte size in males compared to females on micro-computed tomography^46^, while another compared sexes in a non-invasive rat ACL rupture model and reported non-significant trends toward increased OARSI score in females^47^. Notably, sex differences have been described in related, non-surgical studies. For example, male rats with monoiodoacetate-induced arthritis are more sensitive than females to experimental pain-reducing therapies, including exposure to green light-emitting diode strips^48^ and antidepressants^49^. In this study, female animals demonstrated increased mechanical hyperalgesia and more severe histologic cartilage degeneration post-ACLT compared to males. This finding emphasizes the importance of including both sexes in preclinical models of OA. While determining the mechanism of sex-related differences was beyond the scope of this study, tissue injury may have been more severe in females due to a smaller knee joint size, as 12-week Sprague-Dawley female rats were approximately half the size of male rats. With sex being a risk factor for OA in humans^50^, further investigation into sex differences in animals is warranted.

The structural outcomes of lubricin intra-articular injection in female versus male animals following OA induction has yet to be fully investigated. Of note, a previous study investigated delayed rhLub intra-articular injection in female rabbits after induction of osteochondral lesions, and reported increased proteoglycan staining in rhLub-treated joints compared to PBS treatment, as well as trends toward gross improvement of cartilage repair 12-weeks after injury and 8-weeks after rhLub injection^22^. Female rats have been used in early optimization of lubricin injection and in the study of lubricin joint clearance, but further research into functional or structural outcomes in these animals post-injury is lacking^51^. rhLub, like lubricin, functions at the joint surface and, because female animals exhibited greater lesion depth in the cartilage compared to males, rhLub may have been unable to protect against degeneration in female animals due to the more severe structural changes in females relative to males. Previous work investigating lubricin analogs in minipigs post-injury has demonstrated that lubricin is unable to rescue a severe joint disease phenotype post-destabilization^52^. Female rats were smaller than males, and optimization of injection volume (25 µL) prior to this study was performed in male but not female rats^36^. While previous work in uninjured Sprague-Dawley female rats weighing between 200 and 250 g found an optimal knee joint injection volume to be approximately 30 µL^53^, the volume injected may have exceeded the volume of the injured joint capsule, resulting in higher rates of peri-articular leakage and reduced retention of rhLub in females. Additionally, this study was limited by imperfect orientation of histology sections. While most sections were in the frontal plane, several sections were oblique.

Previous purified and recombinant lubricin injectables have shown promise as therapeutics for OA. This bioengineered lubricant inspired by human lubricin (rhLub) maintains the structural components, the N- and C-terminal domains, and the glycosylated mucin-like backbone of native lubricin essential for its anti-inflammatory and anti-adhesive effects^27^. After ACLT, rhLub protected against cartilage and bone-related changes associated with OA development in male rats, but not females. Interestingly, surgical ACLT induced more severe cartilage degeneration and pressure withdrawal sensitivity in female compared to male rats. This work strongly supports sex as a biological variable and emphasizes the importance of future study of OA in both sexes. Overall, the chondroprotective effects of rhLub in rat knees after ACLT provides strong evidence for its potential as an intra-articular therapeutic for mild-to-moderate OA^54^.

## Methods

### Animals

All procedures were approved by the Cornell University Institutional Animal Care and Use Committee (2017-0084), were performed according to national and university regulations, and are reported in accordance with Animal Research: Reporting of In Vivo Experiments (ARRIVE) guidelines. Male and female 10- to 11-week-old Sprague-Dawley rats (*n* = 18 total) were housed in same-sex pairs under a standard 12 h light/dark cycle beginning at 6:00 a.m. The animals moved freely in their cages, were fed a standard commercial diet, were allowed access to tap water, and were provided with cylindrical or rectangular plastic tunnels as enrichment. Means from a previous study^25^ were used to determine effect size, with an *n* = 8 needed for statistical significance. Initially, 10 animals per sex were enrolled to account for possible losses, with one male and one female rat excluded from analysis to yield final group sizes (*n* = 9).

### Study design

Baseline functional outcomes were obtained within 1 week prior to surgery, following a 3-day quarantine and acclimatization period. At 12-weeks of age, anterior cruciate ligament transection (ACLT) surgery was performed bilaterally via an open surgical approach under 2.5% isoflurane anesthesia at 1 L/min O_2_, as previously described^36^. Complete ACLT was confirmed via a cranial drawer test. The joint capsule was closed with 3 − 0 multifilament polyester (Ethibond) and the skin was closed with 4 − 0 monofilament nylon (Ethilon) suture in a simple interrupted pattern, with skin glue (3M, Vetbond) applied to the incision after closure. At the time of surgery, rats were treated with an extended-release formulation of buprenorphine (Ethiqa XR, 0.65 mg/kg) subcutaneously. All animals were able to eat, drink, and ambulate *ad libitum* after surgery. Animals were monitored daily for 1 week following surgery. Sutures were removed 7 days post-operatively, at the time of the first injection.

Beginning 1-week post-ACLT, 25 µL of either phosphate-buffered saline (PBS) or recombinant human lubricin (rhLub, 1.3 mg/mL) was administered intra-articularly via a lateral parapatellar approach with a sterile 0.5” 27 G needle on a 0.5 mL syringe as previously described^36^. All animals received injections bilaterally, with one randomly selected knee receiving the rhLub treatment (*n* = 8 left limbs, *n* = 10 right limbs) and one knee receiving PBS (*n* = 10 left limbs, *n* = 8 right limbs). Injections were performed once weekly for three weeks, a design chosen based on previous literature in the rat ACLT model^36,42^. At the time of the first injection, laterality of intra-articular treatment was determined via coin flip and was blinded to all investigators performing functional measurements.

Functional testing began 1-week post-surgery concurrently with intra-articular injections. Static weightbearing via an incapacitance meter was performed on day 1 post-injection, and mechanical hyperalgesia using pressure application measurement (PAM) was performed on day 2 post-injection. Both tests were performed weekly on the same day of the week and at approximately the same time of day by the same two operators, excepting week 7, for the duration of the study (12-weeks). Functional testing procedures were conducted in a separate room from the housing area. At week 12, animals were humanely euthanized by CO_2_ administered into a standard rat cage at a rate of 10.8 L/min followed by cervical dislocation.

### Bioengineered recombinant human lubricin-inspired glycoprotein (rhLub) production and purification

Lubricin-inspired glycoprotein (rhLub) was produced via suspension culture in Human Embryonic Kidney (HEK)-293 cells, and purified and sterilized as previously described^19^. Briefly, suspension adapted HEK-293 cells (Thermo Fisher Scientific) were co-transfected with an expression plasmid for rhLub, generated by custom gene synthesis and containing cDNA for a human lubricin-inspired bioengineered glycoprotein with 59 tandem repeats of the characteristic lubricin KEPAPTTP repeat sequence. Cultures were grown in a shaking incubator at 37 °C, 5% CO_2_, and 85% humidity, prior to transfer into a wave-mixed bioreactor with a maintained pH of 7.2, temperature of 35°C, and pO_2_ of 60% through online control of the overlay gas mixture and addition of sodium bicarbonate. Neat, rhLub-containing media was harvested by allowing cells to sediment for 1 h at room temperature in the culture vessel, then clarified by depth filtration. The rhLub protein was purified by liquid chromatography in a 3-step chain consisting of cation exchange, hydrophobic interaction, and size exclusion operations. The concentrated product was sterilized using dead-end membrane filtration (0.2 μm pore polyethersulfone membrane), lubricin concentration measured by enzyme-linked immunosorbent assay (ELISA), and diluted to the final concentration in sterile formulation buffer. Individual aliquots of the sterile rhLub were transferred to clean, sterile, pyrogen-free serum vials, sealed with a rubber septum cap, frozen in liquid nitrogen and stored at -80 °C prior to injection.

### Tissue processing and histology

Immediately following euthanasia, muscle was dissected from the femur and tibia of both hindlimbs. Knees were fully extended and secured to a thin wooden rod prior to fixation in 10% neutral-buffered formalin for 3–6 d. Following fixation, samples were decalcified in 10% ethylenediaminetetraacetic acid for 21–33 d until complete decalcification was confirmed by radiography (Faxitron, 60 s exposure at 37 V). Tissues were processed in ascending alcohol concentrations, cleared in xylene, and transected frontally approximately halfway through the joint with the cranial and caudal joint halves placed in separate cassettes prior to paraffin embedding for histological sectioning. After obtaining frontal sections (5 μm) throughout the entire joint, sections of both mid-cranial and mid-caudal aspects of the joint were stained using hematoxylin and eosin (H&E) and Safranin O/Fast Green. To ensure inclusion of consistent anatomic location between sections, slides were selected for scoring by one blinded observer (S.J.W.) based on medial meniscal length (1/3 of the tibial plateau length). Slides were imaged at 20x magnification (Aperio, ScanScope CS2) and scored using the Aperio ImageScope x64 software.

#### Hematoxylin and eosin and safranin-O fast green staining

Slides underwent standard xylene deparaffinization and ethanol rehydration procedures. Following rehydration, slides were air-dried for 30 min and then rinsed twice with clean, distilled water for two minutes. During H&E staining, slides were stained in hematoxylin (Epredia, Cat #7211) for eight minutes, washed under running tap water for one minute, then dipped 1–3 times into acid alcohol (1% hydrochloric acid in 70% ethanol) to remove excess dye. Slides were rinsed in running tap water for ten minutes then stained in eosin-B (Sigma-Aldrich, Cat #861006) for three minutes. For Safranin-O Fast Green staining, slides were stained in hematoxylin for six minutes, then dipped 1–3 times into acid alcohol to remove excess dye and briefly rinsed in running tap water. Slides were stained in Fast Green (EMS, Cat #15500) for five minutes, 1% acetic acid for one minute, Safranin-O (Ward’s Science, Cat #470302-32) for six minutes, and then rinsed in running tap water for five minutes. Independent of stain, slides were then dehydrated in an ascending series of ethanol solutions, rinsed in xylene three times for three minutes each, then coverslipped with Cytoseal 60 mounting medium (Epredia, Cat #831016).

#### Tissue scoring

Medial tibial plateaus of H&E and Safranin-O Fast Green-stained knee sections cut in the frontal (coronal) plane were scored by three blinded observers (S.J.W., S.R.N., A.G.F.), including a boarded anatomic veterinary pathologist (S.R.N.), using criteria outlined in the Osteoarthritis Research Society International (OARSI) guidelines^55^. Oblique sections were included in analysis. Two sections were scored per knee, one taken from the caudal (posterior) half of the joint and one from the cranial (anterior) half, excepting one rat in which the caudal section of one joint was not able to be scored due to histological artifact obscuring the entire medial tibia.

#### OARSI subcomponents

*Tibial Cartilage Loss*: Measurements of cartilage matrix loss were taken at the surface, middle, and tidemark (µm). This measurement was normalized to the total length of tibial cartilage.

*Tibial Cartilage Degeneration*: Total and significant levels of tibial cartilage degeneration were measured. This measurement was normalized to the total length of tibial cartilage.

*Cartilage Degeneration Score*: The cartilage degeneration score accounted for the entire length and depth of cartilage changes, including proteoglycan loss/loss of Safranin-O stain, chondrocyte death, and cartilage matrix loss.

*Average Lesion Depth Ratio*: The depth of each lesion, determined via both matrix loss and other degenerative changes, at three equidistant locations in the cartilage, was measured. This score is a ratio of the lesion to projected cartilage surface and ranges from 0 to 1.

*Bone Score*: The subchondral bone was scored for degenerative changes such as fragmentation of the tidemark and calcification of the bone.

*Osteophyte Score*: If observed, osteophytes were scored and measured in micrometers at the widest part of the osteophyte.

*Total OARSI Score*: Total OARSI score was calculated as the sum of cartilage, bone, and osteophyte scores.

### Static weightbearing and mechanical hyperalgesia

Static weightbearing was assessed via an incapacitance meter (IITC Life Sciences, Cat #600) as previously described^36^. Rats were placed into a chamber atop two force plates. Once rats were positioned with one foot on each force plate, the average weight on each transducer was recorded over a 5 s interval, for a total of 5 trials per rat, per testing day. The percentage of total weightbearing on the rhLub-injected limb was reported for each rat.

Mechanical hyperalgesia was assessed using pressure application measurement (PAM) (Ugo Basile, Cat #38500). Animals were restrained with the left hand while the operator applied pressure at a constantly increasing rate to the medial aspect of the knee using a force transducer attached to the right thumb. Three trials were collected per leg, per testing day. The rate of force applied was approximately 150 g/s until either a withdrawal behavior (physical withdrawal of the limb, vigorous movement of the body, vocalization) or a maximum duration of 5 s or force of 750 mN was attained^56^. The maximum force (mN) and time (s) prior to withdrawal was recorded.

### Statistical analysis

Histological data was analyzed using a linear mixed effects model with fixed effects of sex, treatment, and scorer, a main effect of laterality, and random effect of rat (*n* = 36; 18 limbs per sex). Intraclass correlation coefficients were calculated for each histologic component score. A linear mixed effects model with fixed effects of week, sex, and treatment, a main effect of laterality, and random effect of treatment nested within rat was used to analyze functional testing data. Histological and functional testing data was plotted and visualized to ensure that assumptions of normality of residuals and homogenous variance were met for linear mixed effects models. All data was analyzed at a significance level of α = 0.05 and is represented as mean ± standard error of the mean. Statistical analyses were performed in RStudio 4.3.1.

## Supplementary Information

Below is the link to the electronic supplementary material.

Supplementary Material 1

## Data Availability

The datasets generated and/or analyzed during the current study are available from the corresponding author upon request.
