# Supplementary material for: Lubricin protects against cartilage degeneration following anterior cruciate ligament transection in rats
Source: Sci Rep. 2026 Apr 20;16:18031. doi: 10.1038/s41598-026-45349-9 (PMC13254249; doi:10.1038/s41598-026-45349-9)
Supplement: Supplementary file 1 — Supplementary Material 1 [file 41598_2026_45349_MOESM1_ESM.pdf]

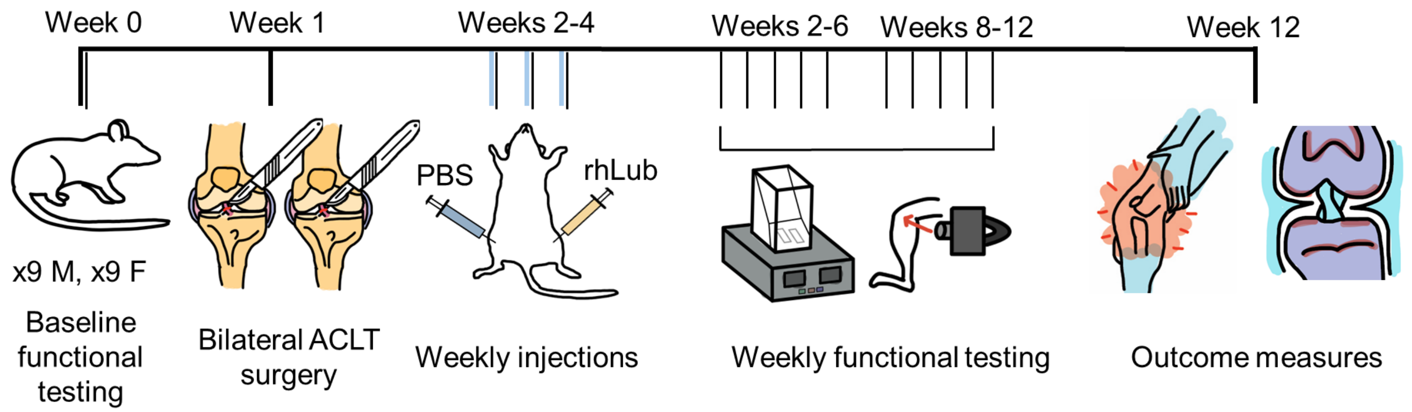

Blue dashes indicate injection timepoint. Thin black dashes indicate functional testing timepoint.

Supplementary Figure S1. Study design overview. ACLT = anterior cruciate ligament transection, PBS = phosphate-buffered saline.

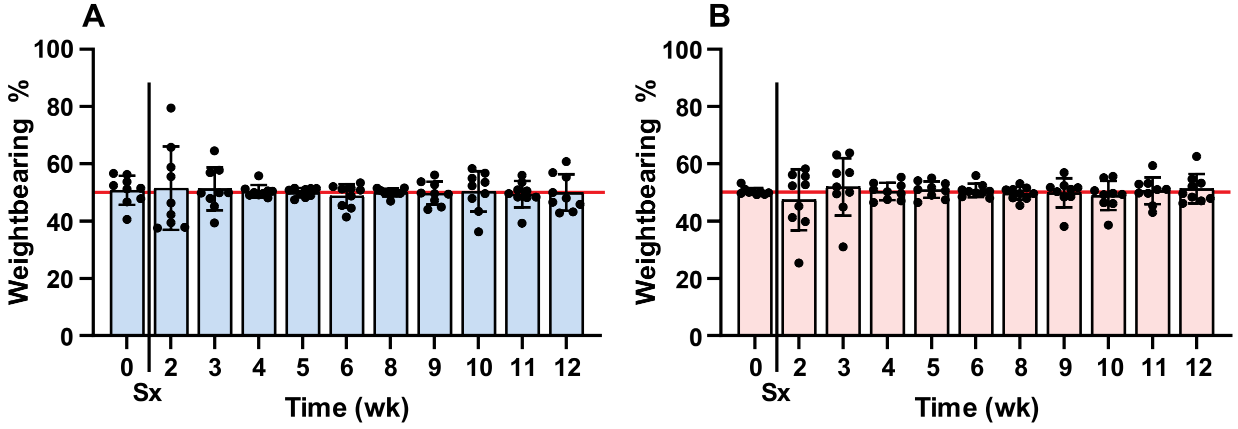

Supplementary Figure S2. There were no adverse effects of rhLub injection in either male **(A)** or female **(B)** rats, with no differences between limbs at any point in the study. Data is represented as weightbearing percentage on the rhLub-treated limb relative to the total weightbearing on both limbs.

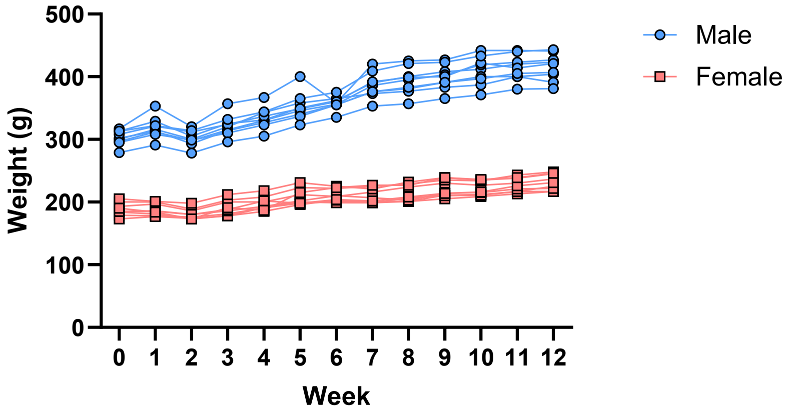

	**Males**		**Females**	
Week	Mean (g)	SEM	Mean (g)	SEM
0	303	4	188	3
1	318	6	188	3
2	302	4	181	3
3	321	6	191	4
4	334	6	197	4
5	352	7	208	4
6	358	4	211	3
7	386	7	211	4
8	393	7	215	4
9	399	6	222	5
10	409	8	222	4
11	414	7	227	4
12	415	7	232	4

Supplementary Figure S3, Supplementary Table S1. Rats of both sexes increased in weight throughout the course of the study, with transient weight loss one week after surgery. This loss was recovered by the second week post-ACLT.

Supplementary Table S2. Withdrawal force on the PBS-treated limb.

	**Males**				**Females**				**Sex comparisons**	
Week	Mean (mN)	SEM	p	% to baseline	Mean (mN)	SEM	p	% to baseline	M-F	p
0	720	20			716	21			4	0.9
2	618	34	0.5	14%	497	42	0.0002*	31%	121	0.02*
3	586	35	0.1	19%	553	28	0.02*	23%	33	0.5
4	694	21	1.0	4%	553	29	0.02*	23%	141	0.008*
5	628	35	0.7	13%	494	21	0.0002*	31%	135	0.01*
6	615	40	0.5	15%	399	28	<0.0001*	44%	216	0.0001*
8	702	16	1.0	2%	432	18	<0.0001*	40%	270	<0.0001*
9	685	28	1.0	5%	423	27	<0.0001*	41%	261	<0.0001*
10	657	29	1.0	9%	420	17	<0.0001*	41%	237	<0.0001*
11	687	33	1.0	5%	414	26	<0.0001*	42%	273	<0.0001*
12	638	38	0.8	12%	454	33	0.0001*	37%	181	0.0007*

* Indicates statistical significance from baseline (within sex) or between sex comparisons.

Supplementary Table S3. Withdrawal force on the rhLub-treated limb.

	**Males**				**Females**				**Sex comparisons**	
Week	Mean (mN)	SEM	p	% to baseline	Mean (mN)	SEM	p	% to baseline	M-F	p
0	699	21			645	43			54	0.3
2	566	42	0.1	19%	510	38	0.1	21%	56	0.3
3	590	28	0.4	16%	525	36	0.3	19%	64	0.2
4	669	29	1.0	4%	621	45	1.0	4%	48	0.4
5	684	21	1.0	2%	504	44	0.1	22%	181	0.0008*
6	675	28	1.0	3%	440	44	0.0009	32%	235	<0.0001*
8	710	18	1.0	-2%	418	48	0.0001	35%	292	<0.0001*
9	658	27	1.0	6%	390	41	0.0001	40%	268	<0.0001*
10	728	17	1.0	-4%	418	35	0.0001	35%	310	<0.0001*
11	710	26	1.0	-2%	462	43	0.005	28%	248	<0.0001*
12	662	33	1.0	5%	505	61	0.1	22%	157	0.003*

* Indicates statistical significance from baseline or between sex comparisons.

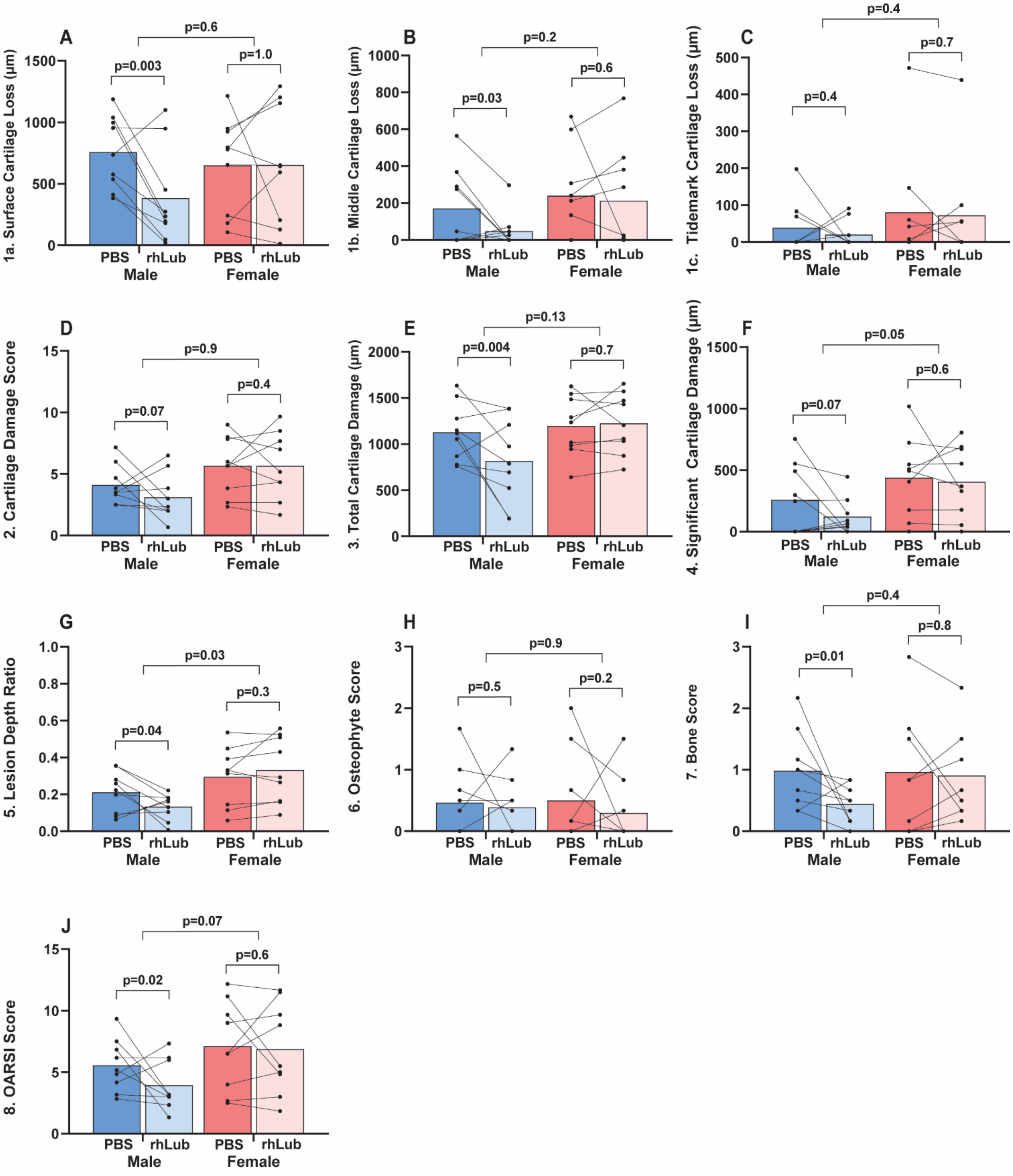

Supplementary Figure S4. Surface and middle cartilage matrix loss was lower in rhLub-treated joints compared to PBS-treated joints in male rats **(A, B)**. Matrix loss at the tidemark and cartilage damage score were unchanged as a function of treatment or sex **(C, D)**. rhLub-treated joints in male rats were protected from total and significant cartilage damage and had shallower lesions than PBS-treated limbs **(E-G)**. Female rats had higher levels of significant cartilage damage and increased lesion depths compared to males, irrespective of treatment **(F, G)**. rhLub protected against degenerative bone changes compared to PBS treatment in male rats **(I)**. Overall OARSI score was decreased in rhLub-treated joints compared to PBS-treated joints in male rats **(J)**. There were no changes in female rats as a function of treatment in any of the OARSI subcomponents or overall OARSI score **(A-J)**.
